# Taking AIM at serious illness: implementing an access to investigational medicines expanded access program

**DOI:** 10.3389/fmed.2023.1287449

**Published:** 2023-10-09

**Authors:** Meghan Morrison Joly, Terri L. Edwards, Rebecca N. Jerome, Alex Mainor, Gordon R. Bernard, Jill M. Pulley

**Affiliations:** Vanderbilt Institute for Clinical and Translational Research, Vanderbilt University Medical Center, Nashville, TN, United States

**Keywords:** FDA expanded access, compassionate use, single patient IND, investigational medicines, access to investigational medicines, regulatory knowledge

## Abstract

When seriously ill patients have exhausted all treatment options available as part of usual care, the use of investigational agents may be warranted. Food and Drug Administration’s (FDA) Expanded Access (EA) pathway provides a mechanism for these patient’s physicians to pursue use of an investigational agent outside of a clinical trial when trial enrollment is not a feasible option. Though FDA has recently implemented processes to significantly streamline the regulatory portion of the process, the overall pathway has several time-consuming components including communication with the pharmaceutical company and the associated institutional requirements for EA use (contracting, Institutional Review Board [IRB], pharmacy, billing). Here, we present our experience building infrastructure at the Vanderbilt University Medical Center (VUMC) to support physicians and patients in pursuing EA, called the Access to Investigational Medicines (AIM) Platform, aligning the needs and responsibilities of institutional stakeholders and streamlining to ensure efficiency and regulatory compliance. Since its launch, the AIM team has experienced steady growth, supporting 40 EA cases for drugs/biologics, including both single patient cases and intermediate-size EA protocols in the emergent and non-emergent setting. As the EA pathway is a complex process that requires expert facilitation, we propose prioritizing EA support infrastructure at major academic medical centers as an essential regulatory knowledge function.

## Introduction

While enrollment in a clinical trial is the preferred method for patients to access investigational medicines, not all patients qualify for clinical trials, for example due to not meeting eligibility criteria (e.g., age restrictions), geographical constraints, and other reasons. For seriously ill patients, FDA allows for more expeditious access to investigational therapies on a case-by-case basis through its EA pathway (also sometimes known as compassionate use). EA allows a patient (or patients) with a serious or immediately life threatening disease or condition to gain access to an investigational drug, biologic or medical device when no comparable or satisfactory alternatives are available (1). Several types of EA are available depending on the number of patients in need, ranging from one individual patient to an intermediate size-patient population (more than one patient) and EA for widespread treatment use (typically used for a large population to bridge the gap between completion of a clinical trial and final approval) (2). Use of the EA pathway has increased in recent years, with 2,261 EA Investigational New Drug (IND) applications and protocols received and 2,248 approved by FDA in 2022 (3).

## Challenge

Navigating the institutional coordination and approvals processes through the EA pathway can be complex, requiring the swift cooperation of numerous stakeholders including the patient, physician and clinical teams, drug company, IRB, FDA, pharmacy and billing teams, among others (2). General steps are outlined below, many occurring in parallel to expedite the process (Supplementary Table 1). First, the treating physician must assess whether the patient qualifies for treatment under the EA pathway outlined in the Code of Federal Regulations (CFR) Title 21 (4). If the patient qualifies, a request is made to the company studying the investigational medicinal product (IMP)—this can either occur through formal pathways (e.g., company request forms submitted through their website) or more informal means (e.g., via email). Company agreement to provide the IMP is the key gating item to EA; if the company does not agree to provide the IMP, which can occur for a variety of reasons (e.g., shortage of supply, may interfere with investigational trials that could support product development or marketing approval), the process cannot move forward. If the company does agree to provide the IMP for EA use, the physician or regulatory facilitators begin gathering necessary clinical information and supporting documents to compile the applications for submission to the appropriate regulatory bodies (i.e., the FDA and IRB). Required documents include a treatment plan that outlines rationale, previous human experience with the IMP, dosing, and an informed consent/assent document. For single patient EA submissions to the FDA submission, FDA 3926 form is required. If a contract is required, this process is also started, and the timing for review and execution varies depending on institutional and company policies. In some cases, an executed agreement is required for the company to provide the letter of authorization (LoA) allowing FDA to cross-reference their IND in support of the current EA treatment or to provide the Investigator’s Brochure (IB).

When all the required documents are completed or obtained, submissions to the IRB and FDA are made. IRBs will typically review in an expedited manner and FDA response times are usually swift, varying by reviewing department. In 2019, FDA announced the launch of the Oncology Center for Excellence (OCE) Project Facilitate, a pilot program to assist oncology providers in requesting access to investigational therapies for patients with cancer through EA pathway and to streamline the regulatory authorization process. In the year following its launch, Project Facilitate supported 640 single patient IND requests (5) and continues to serve an important role in streamlining EA support. The Center for Disease Evaluation and Research (CDER) and Center for Biologics Evaluation and Research (CBER) have also streamlined the submission/review process for non-emergency EA applications through creation of the CDER NextGen portal. Once all the necessary approvals are obtained, the physician and team coordinates shipping with the manufacturer and dispensing and storage details with the local pharmacy teams and the patient is treated. Finally, after treatment is initiated, treating physicians, as IND sponsors, are required to gather and report safety information, including any adverse reactions, and report to the appropriate regulatory bodies. IND annual reports are also required to be submitted within 60 days of the date the IND became active, activities that often fall outside of the scope of traditional clinical responsibilities.

Beyond the logistical complexities involved in the EA application process described above, there are additional nuances for each case depending on the individual medication including if the medication is a drug or biologic, the route of administration (e.g., infusion or pill), treatment duration (e.g., one time treatment, short term treatment or ongoing treatment), among other factors, with added implications for institutional processes including pharmacy processes.

## Solution

While EA is not considered research, the complexities of the EA process are similar to that of clinical research. Academic medical centers are poised to benefit from their extensive research infrastructure, research experience and ongoing institutional regulatory expertise. Leveraging these strengths and adapting them for EA use is a prime example of how facilitative infrastructure can help integrate research experiences to inform clinical practices. Academic medical centers, including those participating in the NIH-funded Clinical Translational Science Award (CTSA) program, are particularly well-suited to develop and leverage the strengths of this fundamental regulatory knowledge function. Furthermore, the traditional clinical care enterprise at an institution may be less incentivized to develop expertise and familiarity with these types of approval mechanisms due to their relatively rare use. In contrast, existing CTSA program familiarity with a variety of FDA regulatory processes paired with a deep understanding of and expertise with the institutional teams and processes required (IRB, contracting, billing, pharmacy, etc.) can prove beneficial, giving institutions and investigators a strong foundation from which to obtain drug approvals when the need arises (6).

**Figure 1:**
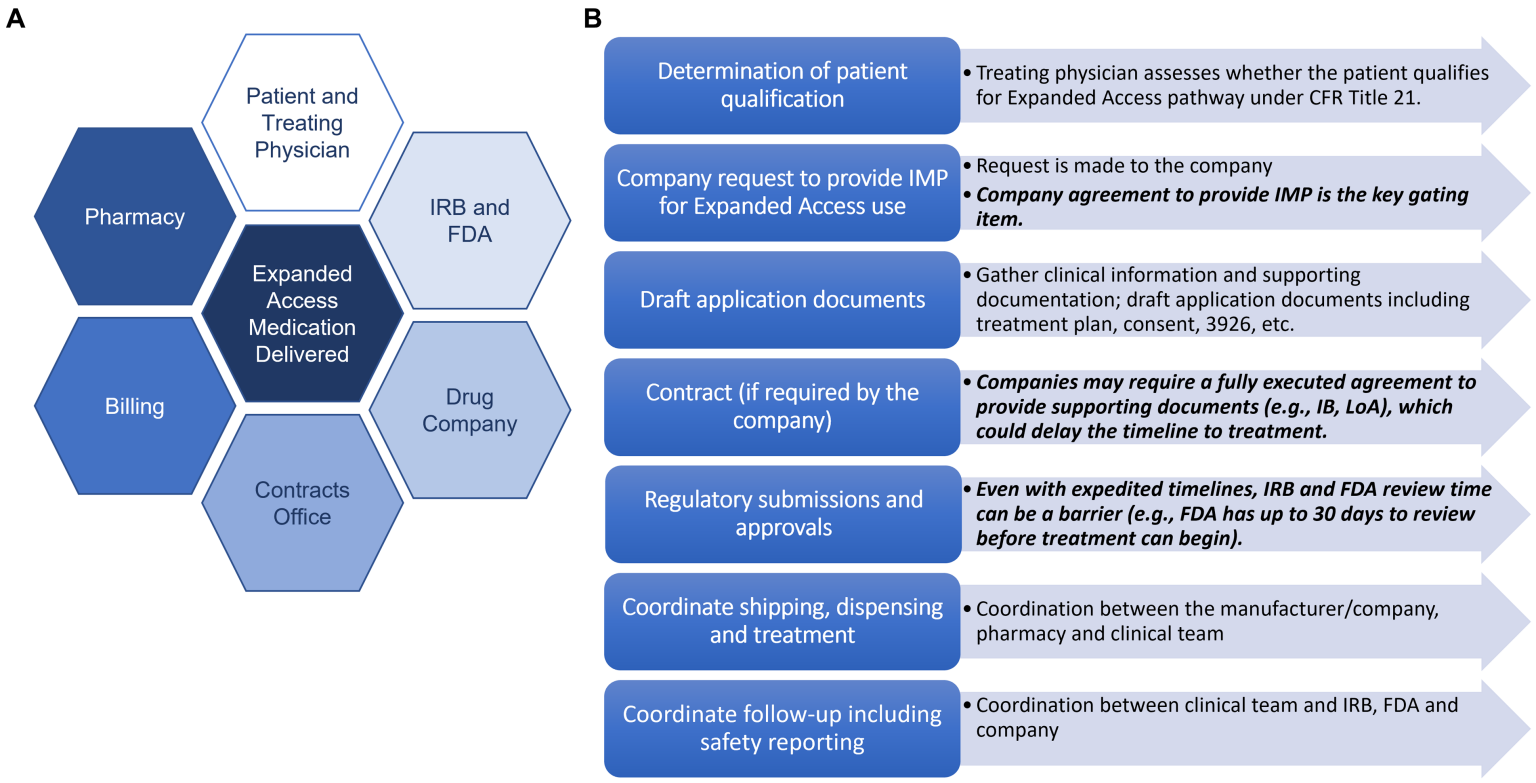
Key stakeholders and steps in the EA process. **(A)** Key stakeholders in the expanded access process. **(B)** Key steps in the expanded access process with potential barriers listed in *black.*

Identifying the need for a more formalized infrastructure at Vanderbilt University Medical Center (VUMC) to support patients and physicians in navigating the complex EA process, the Vanderbilt Institute for Clinical and Translational Research (VICTR) initiated a planning and information gathering phase in 2015, engaging in extensive conversations with key stakeholders (Figure 1A) and developing a set of recommendations for streamlining institutional EA processes (7). Building upon our initial ideas, we established the Access to Investigational Medicines (AIM) platform in 2016 to improve efficiency and ameliorate barriers to EA. Here, we report on our platform’s use and refinement over its first several years since implementation.

## Implementation/methods

Supported by the NIH-sponsored CTSA, the AIM platform provides support for physicians at VUMC who want to pursue access to investigational therapies for their patients through FDA’s EA pathway. The AIM team employs diverse subject expertise spanning regulatory processes (compliance with FDA and IRB requirements), effective informed consent, project management, billing, and contracting, to guide the physician, patient, and drug company team through the EA process (Figure 1B). The AIM team facilitates communication and provides support through all phases of the EA process, from initial inquiry to FDA/IRB application and approval, to delivery of the investigational product and required safety reporting, tailoring support to the physician/clinician’s individual needs. Since supporting our first case in 2018, the team has supported 40 EA cases for drugs/biologics at VUMC (Supplementary Table 2) and has also consulted with multiple institutions external to VUMC for how to best approach EA case support at their institutions.

## Outcomes

**Figure 2:**
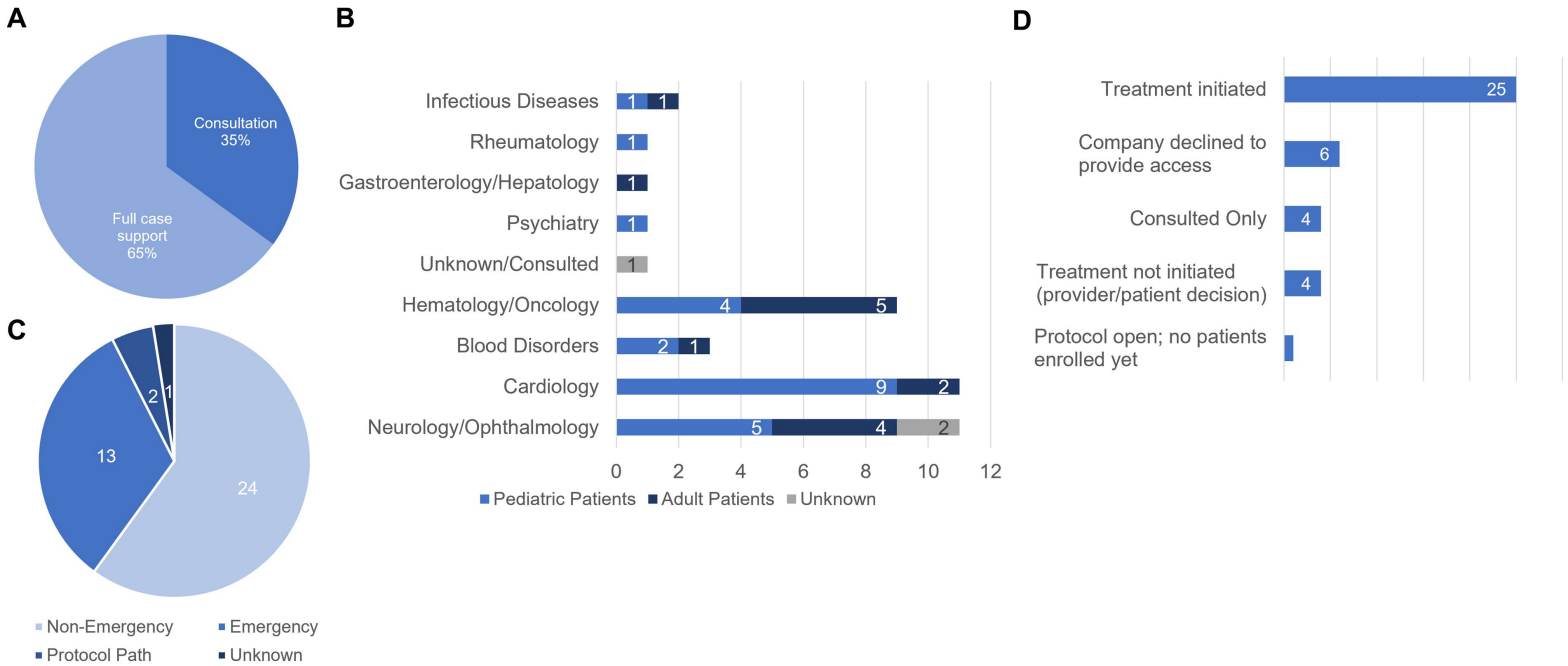
Characteristics and statuses of cases supported by the AIM team. **(A)** Breakdown of the type of support provided (% of total cases supported). We provided consultation on 14/40 cases (35%) and full case support for 26/40 (65%) cases. **(B)** Distribution of patient populations by disease area and age group. **(C)** Designations of cases supported by AIM. **(D)** Treatment statuses of AIM supported cases. For consulted cases, the AIM team provided support but did not track the outcome. Of the 4 cases for which treatment was not initiated, the breakdown is as follows: (1) received all approvals but the family decided not to further pursue, (2) the physician decided to pursue off-label use of the medication in powder form, (3) the patient died prior to initiating the Expanded Access process, and (4) approvals received but provider elected not to initiate treatment due to patient’s condition being stable.

The case support we have provided to date has ranged from consulting with clinical teams on specific pieces of the EA pathway (14/40 cases) to full case support (26/40; Figure 2A). Each of the cases reported here involved patients who met all the eligibility requirements for pursuing the EA pathway. The cases we have supported span diverse clinical areas including Cardiology, Neurology/Ophthalmology, and Hematology and Oncology in both adult and pediatric populations (Figure 2B). Neurology/Ophthalmology and Cardiology represent our most supported clinical area with 11 cases facilitated for patients with Neurology/Ophthalmology-related indications and 11 facilitated for Cardiology-related indications. Supported cases also encompass both single patient emergency and non-emergency designations as well as one intermediate size EA protocol and a larger EA protocol to bridge the gap from treatment to medication approval for commercial use (Figure 2C).

Treatment status for each of the 40 cases we supported are found in Figure 2D. In 15% (6/40) of cases we supported, access to the investigational agent was not granted by the manufacturer and therefore the case did not move forward. Reasons provided include supply issues and lack of supporting data in the population for the requested patient. In 62.5% of cases (25/40), the manufacturer agreed to provide the IMP for EA use, all required approvals were received, and treatment was initiated. In one case (1/40, 2.5%), we successfully worked with the treating physician to open an intermediate size EA protocol to enroll patients who have exhausted all treatment options and may benefit from the investigational agent in an emergent situation, however, no patients have been enrolled or been treated to date. In 10% (4/40) of cases, our support included consulting on the process for fellow colleagues and therefore we did not track the outcome of the case. In 10% (4/40) of cases, treatment was not initiated for a variety of reasons. In one case, the family decided not to pursue treatment with the investigational medicine. Another patient unfortunately passed away before the EA process could be initiated. In one case, the provider decided to pursue off label use of the medication in a different form [tablet vs. powder for reconstitution]. Finally, in one case, the provider decided not to initiate treatment because the patient’s condition stabilized.

## Key lessons learned

1. Early and frequent communication with stakeholders is critical.
2. Proactive interaction with the pharmacy team is key.
3. It is crucial to liaise with the billing compliance team to ensure standard of care charges are billed to the patient/their insurance.
4. Open communication with the IRB is essential for alignment and success, as specific Expanded Access scenarios/uses may have different requirements or issues to be addressed (e.g., require prospective IRB review for repeat emergency use cases of the same medication).

## Discussion

While clinical trials are the mainstay for patient access to investigational medicines, not all patients qualify for clinical trials. In these situations, FDA’s EA pathway plays a key role in bridging this gap. Here, we describe our implementation of an EA support infrastructure at VUMC called the AIM platform and report on our experience supporting 40 EA drug/biologic cases including both single patient cases and EA protocols. In our experience, EA regulatory approvals are typically swift with key stakeholders incorporating procedures for expediting processes (e.g., rapid FDA approval for oncology cases submitted through Project Facilitate, expedited review through our institutional IRB), however, work is ongoing within the AIM platform to further refine processes. For example, VUMC recently established an EA Optimization Working Group to continue to align and streamline institutional processes with key stakeholders including contracting, pharmacy teams, billing, and the IRB among others. Through this working group, we identified the significant role the billing compliance team plays in the EA process, applying the appropriate billing codes to ensure standard of care charges to bill to the patient/their insurance. We are also working closely with the inpatient and outpatient pharmacy teams across the institution to further develop EA planning and standard processes and ensure the appropriate workflows and standard operating procedures (SOPs) are in place to support single patient cases efficiently and within all local policies and laws. Importantly, we note that these types of institutional efforts have led to increased word-of-mouth sharing of the services the AIM team provides which has resulted in increased requests for our support across the institution.

In our experience, the primary barrier to successfully pursuing treatment through FDA’s EA pathway remains the inability or unwillingness of companies to provide the investigational product for EA use (occurring in 15% [6/40] of our cases). Level of involvement in EA varies by company. Though the 21st Century Cures Act requires companies with therapies in Phase 2/3 clinical testing to publicly disclose their policies for evaluating and responding to EA requests (8), a recent analysis revealed that compliance is low, particularly among private companies (9), and despite the requirement to post policies, there is no mandate for companies to provide medications for EA use.

To add further complexity, the Right to Try (RTT) act, which was signed into law on May 30, 2018, provides a second parallel pathway for patients with serious/life-threatening diseases or conditions who have tried all approved therapy options to access investigational therapies (not devices). Though RTT exists, this pathway is redundant, less regulated, and scarcely used (10, 11). The EA pathway continues to function as intended, serving as the key pathway to help patients gain access to investigational agents outside of the traditional clinical trial space and in dire circumstances.

Other institutions are also addressing EA challenges in various ways. A 2011 survey reported that 55% (11/15) of CTSA hubs who responded reported having formal infrastructure available to support with traditional INDs in addition to EA applications (12) and our anecdotal evidence suggests this number has increased since 2011. To complement the regulatory expertise provided at individual CTSA hubs, the National Center for Advancing Translational Sciences (NCATS) recently funded a U01 Collaboration and Innovation award called TEAMSS (Transforming EA to Maximize Support and Study), a partnership led by the University of Michigan in collaboration with Duke University, the University of Rochester, and the University of Texas Southwestern. TEAMSS is focused on the development and dissemination of best practices for creating an integrated and nationwide approach to EA (13). TEAMSS recently completed a national landscape analysis at 47 Academic Medical Centers, with a focus on those with CTSAs, finding that the majority of centers (43/47) reported using single patient EA and 89% reported a support infrastructure for single patient EA cases, however, only roughly half of these centers reported central tracking of EA requests and gaps remain in providing comprehensive support at the centers (14).

Given that EA is a complex and often arduous process that requires expert facilitation, we view providing formal EA support as an essential regulatory knowledge function and advocate for prioritizing this type of infrastructure at major academic medical centers. Even with strong infrastructure support, it should be acknowledged that companies take on substantial risk when agreeing to provide their investigational medications for EA and in some situations, this poses a significant barrier to entry. We envision creating a central clearinghouse (7) to support registration of companies that have offered their investigational medicines for appropriate EA purposes. Such a central clearinghouse would help promote efficiency and transparency and could also integrate seamlessly into Reagan Udall’s existing model of support (15), allowing us to collectively build upon key information and resources to streamline EA and ultimately deliver potentially lifesaving treatment to patients more quickly.

## Data availability statement

The original contributions presented in the study are included in the article/Supplementary material, further inquiries can be directed to the corresponding author.

## Author contributions

MJ: Conceptualization, Data curation, Investigation, Project administration, Writing – original draft, Writing – review & editing. TE: Conceptualization, Funding acquisition, Methodology, Project administration, Resources, Supervision, Writing – review & editing. RJ: Conceptualization, Investigation, Methodology, Project administration, Resources, Supervision, Writing – review & editing. AM: Conceptualization, Investigation, Project administration, Writing – original draft, Writing – review & editing. GB: Conceptualization, Funding acquisition, Methodology, Resources, Supervision, Writing – review & editing. JP: Conceptualization, Funding acquisition, Methodology, Resources, Supervision, Writing – review & editing.
