# Supplementary material for: Taking AIM at serious illness: implementing an access to investigational medicines expanded access program
Source: Front Med (Lausanne). 2023 Oct 9;10:1287449. doi: 10.3389/fmed.2023.1287449 (PMC10590908; doi:10.3389/fmed.2023.1287449)
Supplement: Supplementary file 1 [file Table_1.DOCX]

**Supplemental Table 1. The role of each party involved in the EA process**(1)

| **Party involved** | **Role** | **Approval required** |
| --- | --- | --- |
| Patient | Serious or life-threatening disease or condition, not eligible for clinical trial enrollment, and no comparable or satisfactory therapies are available | Yes; provides informed consent to receive treatment |
| Treating physician/IND sponsor | Determines patient qualification for EA, works with patient on available treatment options, agrees to oversee the patient’s treatment; works with the IMP developer, holds the IND (for many EA request types) and is responsible for patient care and safety reporting | Yes; treating physician must agree to oversee the patient’s care and serve as the responsible party on the FDA application including adhering to safety reporting requirements |
| Company/IMP Manufacturer | If agrees to support the request, provides the IMP and supporting materials (e.g., Letter of Authorization to cross-reference their IND, IB, dispensing training and instructions) | Yes; must agree to provide the IMP |
| FDA | Reviews the EA request and determines if the treatment may proceed; annual review of safety reporting | Yes; *Emergency* – hotline call center, obtain emergency IND (eIND), email approval, follow up with PDF letter; full submission required within 15 business days of telephone authorization  *Non-emergency* – full submission/approval required before treatment can begin |
| Contracting | Institutional contracts office reviews and negotiates the contract with the company/IMP manufacturer | Depends; varies by company and institutional requirements |
| IRB | The IRB has overall responsibility for ensuring institution's regulatory compliance including with EA regulations. Reviews the EA request and determines if the treatment may proceed (prospective review not required for emergency cases). | *Yes; Emergency* – in some cases, prospective IRB review is required (e.g., if drug company requires before drug is shipped); in most cases, however, IRB approval within 5 days of emergency use is required. IRB emergency hotline required for all cases though.  *Non-emergency* –prospective review of materials including treatment plan, informed consent, necessary FDA forms, etc., all required to be reviewed and approved by the IRB |
| Regulatory support infrastructure | Provides support through all the phases of the application process (from inquiry to FDA application to delivery of the IMP), facilitates communication among key stakeholders, develops SOPs and documentation; support is tailored to the needs of the treating physician for each case | N/A |
| Pharmacy (clinical pharmacy or investigational pharmacy) | Confirms medication details and administration instructions, dispenses medication, manages storage, resupply, destruction of unused supply per company or institutional policies | N/A |

**Supplemental Table 2. Therapeutics successfully obtained for EA Use and their manufacturer**

| **Therapeutic name** | **Manufacturer** | **EA Use(s)** |
| --- | --- | --- |
| Levosimendan (Simdax) | Tenax Therapeutics | Heart failure/dysfunction |
| GS010 (Lumevoq) | GenSight Biologics | Leber’s Hereditary Optic Neuropathy (LHON) |
| Hemopure | HbO2 Therapeutics | Severe anemia (when blood transfusion is not an option) |
| Evinacumab | Regeneron | Homozygous Familial Hypercholesterolemia (HoFH) |
| ACER-001 | Acer Therapeutics | Corticobasal degeneration |
| Lirentelimab (AK002) | Allokos Inc. | Eosinophilic gastritis and/or eosinophilic duodenitis |
| Trametinib | Novartis | Severe hypertrophic cardiomyopathy, Noonan syndrome, neurofibroma |
| Tazemetostat | Epizyme Inc. | Atypical teratoid rhabdoid tumor (ATRT) |
| Tipifarnib | Kura Oncology | Metastatic epithelial-myoepithelial carcinoma |
| Tremelimumab | AstraZeneca | Hepatocellular Carcinoma (HCC) |
| MAS825 | Novartis | Refractory systemic idiopathic rheumatoid arthritis, macrophage syndrome, and interstitial lung disease |
| Clofazimine | Novartis | Mycobacterial infections |
| Belantamab mafodotin (Blenrep) | GlaxoSmithKline (GSK) | Refractory multiple myeloma |
| Convalescent Plasma | Vanderbilt University Medical Center | COVID-19 |
| VAL-083 | Kintara Therapeutics | Glioblastoma (GBM) |

1. Commissioner O of the. Expanded Access | Information for Physicians. FDA [Internet]. 2020 Sep 24 [cited 2022 Jul 28]; Available from: https://www.fda.gov/news-events/expanded-access/expanded-access-information-physicians
